# Biocompatibility and bioactivity of bioceramic endodontic sealer: NeoSealer Flo

**DOI:** 10.1590/1678-7757-2025-0284

**Published:** 2025-09-22

**Authors:** Evelin Carine Alves SILVA, Jéssica Arielli PRADELLI, Guilherme Ferreira da SILVA, Paulo Sérgio CERRI, Mario TANOMARU-FILHO, Juliane Maria GUERREIRO-TANOMARU

**Affiliations:** 1 Universidade Estadual Paulista Faculdade de Odontologia Departamento de Odontologia Restauradora Araraquara SP Brasil Universidade Estadual Paulista (UNESP), Faculdade de Odontologia, Departamento de Odontologia Restauradora, Araraquara, SP, Brasil.; 2 Unisagrado Departamento de Odontologia Bauru SP Brasil Unisagrado, Departamento de Odontologia, Bauru, SP, Brasil.; 3 Universidade Estadual Paulista Faculdade de Odontologia Departamento de Morfologia Araraquara SP Brasil Universidade Estadual Paulista (UNESP), Faculdade de Odontologia, Departamento de Morfologia, Araraquara, SP, Brasil.

**Keywords:** Calcium silicate, Endodontics, Materials testing

## Abstract

Bioceramic materials are developed based on their biological properties and bioactive potential. NeoSealer Flo (NeoFlo, NuSmile, Houston) is a ready-to-use bioceramic endodontic sealer.

**Objective:**

The aim of this study was to evaluate the biocompatibility and bioactivity of NeoFlo compared to Bio-C Sealer (BC, Angelus) and AH Plus (AHP, Dentsply).

**Methodology:**

The tissue reaction induced by materials in rat subcutaneous tissues was assessed at seven, 15-, 30-, and 60-days post-implantation (n=6/group). The number of inflammatory cells (ICs), fibroblasts, and osteocalcin (OCN)-labelled cells were recorded. Amorphous calcite was identified using the von Kossa method and polarized light. The data were evaluated using two-way ANOVA followed by Tukey’s test, with a 5% significance level. OCN data were submitted to the Kruskal-Wallis test, with Dunn, Friedman, and Nemenyi post-hoc tests.

**Results:**

NeoFlo capsules showed higher number of IC than BC and AHP (p<0.05) in all periods, with a reduction over time, and was considered moderate at 60 days. Moreover, significant reduction in the number of IC and an increase in the fibroblasts was accompanied by an increase in the amount of collagen in the capsules around all materials over time. Immunoexpression of OCN was only observed in the capsules of NeoFlo and BC, but the capsules of BC showed the highest values in all periods (p<0.05).

**Conclusion:**

In the present study, NeoFlo showed lower biocompatibility than BC, however, NeoFlo shows bioactivity in connective tissue.

## Graphical abstract

**Figure:**
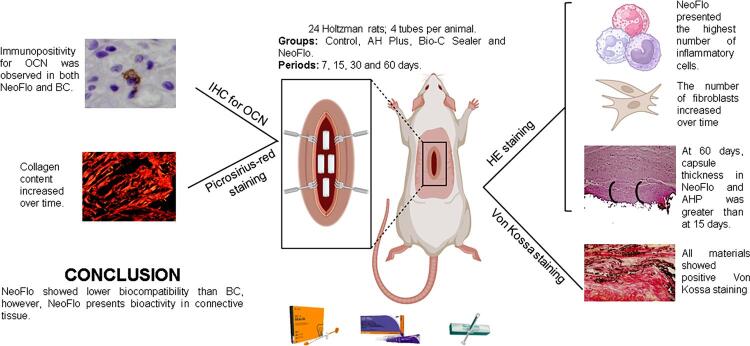

## Introduction

Calcium silicate materials promote calcium silicate hydrate and calcium hydroxide, which have wide clinical applications including ready-to-use materials setting with moisture in dentin and adjacent tissues.^1-3^ New endodontic sealers based on calcium silicate are developed because of their excellent biological properties and bioactivity.^4^

NeoSealer Flo (NeoFlo, NuSmile, Houston, TX, USA) is a ready-to-use bioceramic root canal sealer.^1^ According to the manufacturer, it has hydroxyapatite formation ability, biocompatibility, dimensional stability, and antimicrobial activity. NeoFlo has adequate physicochemical properties and releases biologically active ions, such as calcium and phosphate. Clinically, it obtained a success rate of obturated cases of 96.5% versus 94.9% when compared to EndoSequence BC.^5^ The addition of cetrimide to NeoSealer Flo promoted a greater inflammatory reaction, but the reduction of inflammatory cells and rearrangement of connective tissue suggests that NeoSealer Flo and the associations with cetrimide are biocompatible and have bioactive potential.^6^

Bio-C Sealer (BC, Angelus, Londrina, Paraná, Brazil) is a ready-to-use bioceramic endodontic sealer available in a single syringe that has biocompatibility and bioactive potential^2^. BC has alkalinization ability, adequate flow, radiopacity, and volumetric stability.^7^

AH Plus (AHP, Dentsply DeTrey GmbH, Konstanz, Germany) is an endodontic sealer based on epoxy resin with zirconium oxide and calcium tungstate, which has greater radiopacity than TotalFill BC Sealer.^8^ AHP promotes an initial inflammatory reaction that decreases over time, and it is considered biocompatible. However, AH Plus does not have bioactivity.^8^

The analysis of biomaterials implanted into subcutaneous connective tissue is a controlled methodology recommended by the Fédération Dentaire Internationale and the International Standard-ISO as an indicator for comparing the irritability level of dental materials. This enables accurate assessment of the reactions caused by the material, providing a description of the type, extent, and duration of the lesions. Furthermore, studies using the implantation of biomaterials in subcutaneous tissues demonstrate significant results regarding the effects of different endodontic materials on inflammation and biomineralization markers. The investigation of tissue reactions and bioactive potential of bioceramic endodontic sealers is crucial to understand the mechanisms underlying periapical tissue repair and regeneration, and to enhance the clinical effectiveness of these materials.

Biocompatibility is the ability of a material to promote a biological response with reduced inflammatory reaction leading to structural and functional tissue repair. A bioactive material can interact with living tissue, resulting in the formation of an apatite layer at the material-tissue interface^2^. Biocompatibility and bioactivity of bioceramic endodontic sealers are expected to promote periapical tissue repair after endodontic treatment.^1,9,10^The von Kossa method evaluates the deposition of calcium based on the assessment of calcium precipitation. The induction of mineralized deposits may be assessed by immunohistochemistry to detect proteins such as osteocalcin, which is a peptide secreted by osteoblasts during bone formation.^2^

This study aimed to evaluate the biocompatibility and bioactivity of NeoFlo bioceramic sealer in comparison to Bio-C Sealer and AH Plus. The null hypothesis was that the difference between the composition of the materials would not interfere with the tissue reaction induced by the different sealers.

## Methodology

The regulations for animal use were strictly followed in accordance with the directives of the United Kingdom Animals Use Act 1986. The research protocol was approved by the Ethical Committee for Animal (Protocol # 19/2021). Twenty-four male *Holtzman* rats (*Rattus norvegicus albinus*) were distributed into four groups (n=6/group). The sample size was based on previous studies^2,11,12^ to detect a 50% difference between the experimental groups with control group, with an assumed variability of 20%, a test power of 90%, and an alpha error of 0.05 to identify a significant difference. Therefore, a minimum of five rats per group was required at each time point, and an additional rat was included in each group to account for potential animal loss. Since there was no animal loss, six rats per group were analyzed.

**Figure 1:**
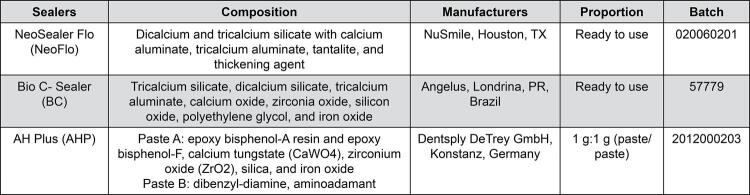
Endodontic Sealers, compositions, manufacturers, and proportion.

Figure 1 shows the materials evaluated, their manufacturers, compositions, and proportions. Polyethylene tubes (Embramed Ltda., São Paulo, Brazil) were filled with one sealer or left empty (control group). The animals were anaesthetized with ketamine hydrochloride (80 mg/kg, Virbac do Brasil Indústria e Comércio Ltda., São Paulo, SP, Brazil) and xylazine chloride (8 mg/kg, União Química Farmacêutica Nacional S/A, São Paulo, SP, Brazil). Subsequently, four tubes were implanted in the subcutaneous connective tissue of the dorsal region of the animals, using a rotation system, enabling the variation in the position of each implanted tube (including the tubes filled with sealers and the control group). This approach enabled an equitable and randomized distribution of the implants. After seven, 15, 30, and 60 days, the animals were euthanized with anesthetic overdose and the implants with adjacent tissues were removed. Periods of seven, 15, 30, and 60 days were used to evaluate the dynamics of the tissue response, enabling analysis of the different phases of the inflammatory and repair processes. The acute phase of the inflammatory reaction is observed at seven days, characterized by intense recruitment of inflammatory cells, particularly neutrophils, and macrophages, representing a period for evaluating the initial irritant potential of the materials. The transition between acute and chronic inflammation begins at 15 days and is marked by a reduction in inflammatory cell infiltration and an increase in fibroblast activity, which indicates the beginning of tissue organization. The capsules around the implants are characterized by a reduced presence of inflammatory cells in contrast to the fibroblast population at 30 days, leading to the formation of dense (fibrous) connective tissue. Finally, after 60 days, it is possible to assess whether the complete resolution of the inflammatory reaction has occurred with the presence of capsules displaying the typical structural components of healthy connective tissue (fibroblasts and collagen fibers). Thus, 60 days are essential to confirm the long-term biocompatibility of the materials.^12^

After seven, 15, 30, and 60 days, the implants and adjacent tissues were removed and immersed for 72 hours in a 4% formaldehyde buffered with 0.1M sodium phosphate at pH 7.2. The specimens were dehydrated, cleared, and embedded in paraffin after fixation, and longitudinal sections (6 μm thick) were obtained. Non-serial sections were stained with hematoxylin-eosin (H&E) to estimate the capsule thickness, and the number of inflammatory cells and the fibroblasts in the capsules.

### Thickness of capsules

The thickness of the capsules (TC) was estimated in three H&E-stained non-serial sections in each implant, with minimal distance between the sections of 120 µm. Capsule thickness was estimated in the middle portion from its surface adjacent to the material to its boundary with adjacent tissues.^2,11,13,14^

### Numerical density of inflammatory cells and fibroblasts

The number of inflammatory cells (IC) and fibroblasts (Fb) was evaluated using the Image-Pro Express 6.0 Olympus program. The numbers of IC and Fb were estimated in H&E-stained three non-sections (minimal distance between sections was 120 µm) in each implant. A standard area of 0.09 mm^2^ of the capsule adjacent to the opening of the implanted tubes was captured with objective lens at ×40 magnification (final magnification: ×695 magnification) in each section. For each implant, the number of ICs (including neutrophils, lymphocytes, plasma cells, and macrophages) and Fb was determined from three sections, totaling a standardized area of 0.27 mm^2^ per implant. At the end, the mean value per group and time point was calculated.

After obtaining the IC number, the inflammation reaction intensity was classified according to the following parameter:^11-13^ mild inflammatory reaction (capsule containing until 25 IC/field), moderate inflammatory reaction (capsule containing from 26 until 125 IC/field), and severe/intense inflammatory reaction (capsule containing over 125 IC/field).

### Content of collagen in the capsules

Three non-serial sections per specimen were stained with picrosirius red solution and analyzed under polarized light (BX51, Olympus) to evaluate the amount of birefringent collagen.^11,12,14^ This was computed using image analysis software (ImageJ; National Institutes of Health; Bethesda, USA) and was estimated considering the standardized hue definitions: red/orange (2‒23 and 230‒256), yellow (39‒51) and green (52‒128). The amount of birefringent collagen was calculated and expressed as the percentage of the number of pixels occupied by collagen.

### Immunohistochemical detection of OCN

Sections were incubated with rabbit anti-OCN antibody (1:150, code SAB1306277; Sigma-Aldrich, St Louis, MO). The sections were then incubated in streptavidin-biotin kit (Universal Dako LSAB, K0675) after 16 hours in a humidified chamber. After buffer washes, peroxidase activity was revealed by 3,3’-diaminobenzidine chromogen (ImmPACTTM DAB) and sections were counterstained with Carazzi’s hematoxylin. The sections were incubated with non-immune serum instead of the primary antibody as a negative control. The number of immunopositive cells was calculated using an image analysis program (Image-Pro Express 6.0, Olympus, Tokyo, Japan), and the number of immunopositive cells/mm^2^ of the capsule was obtained for each implant.

### The von Kossa reaction and analysis under polarized light

The sections were incubated in an aqueous solution containing 5% silver nitrate under an incandescent lamp (100 watts) for one hour. After incubation, the sections were rinsed with distilled water and immersed in 5% sodium thiosulfate for five minutes. They were then washed again in distilled water for five minutes and submitted to the picrosirius-red method. Birefringent structures in the capsules were evaluated using unstained sections analyzed under polarized light (BX51, Olympus, Tokyo, Japan).^14^

### Statistical analysis

With the aid of the Sigma Stat 2.0 program (Jandel Scientific, Sausalito, CA, USA) the data were evaluated by two-way ANOVA followed by the Tukey test, with a significance level of *p*≤0.05. Osteocalcin data were submitted, for comparison between groups, to the Kruskal-Wallis non-parametric test and Dunn’s post hoc multiple comparison test, and then to Friedman’s test and Nemenyi’s post hoc test for analysis over time.

## Results

### Morphological findings and thickness of capsules (TC)

**Figure 2:**
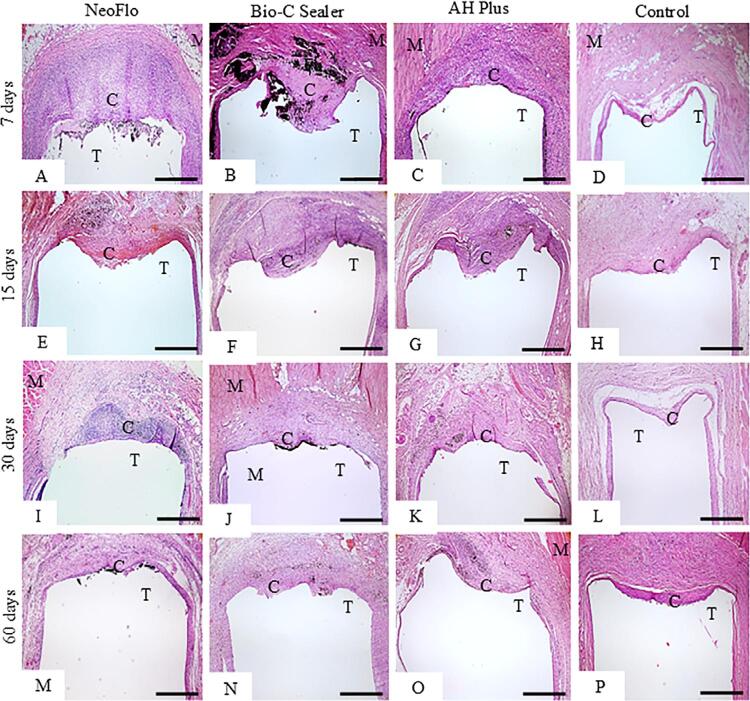
Photomicrographs show a general view of capsules (C) adjacent to the opening of the tubes implanted (T). At seven and 15 days, thick capsules (C) containing numerous inflammatory cells are seen around the implanted materials (Figures 2A-2C and 2E-2G) in comparison with CG specimens (Figures 2D and 2H). At 60 days, well-defined capsules are seen around all specimens (Figures 2M-2P). M, muscle tissue. Bars: 710 μm.

The analysis of the capsules in the H&E-stained sections (Figures 2A-2P) revealed that the materials induced an inflammatory reaction in the connective tissue in close juxtaposition to the opening of polyethylene tubes, mainly at seven and 15 days (Figures 2A-2C and Figures 2E-2G). Thick capsules were adjacent to the implanted materials at 30 days (Figures 2I-2K). The implanted materials were surrounded by well-defined capsules containing fibroblasts and collagen fibers after 60 days (Figures 2M-2O). In contrast, discrete inflammatory reaction was observed in the capsules around the CG specimens at all periods (Figures 2D, 2H, 2L and 2P).

As shown in Table 1, there was no significant difference in the TC between NeoFlo and AHP, while BC showed the highest value at seven days. After 15, 30, and 60 days, there was no significant difference between NeoFlo and BC specimens. The AHP specimens showed greater TC than in NeoFlo, BC, and CG specimens at 60 days. Moreover, there was no significant difference among NeoFlo, BC, and CG at 60 days (*p*>0.05). A significant reduction in the TC was seen in all specimens over time. However, the mean TC values were still greater than 150 µm in the NeoFlo and AHP specimens at 60 days.

**Table 1:** Thickness of the capsules (TC), number of inflammatory cells (IC), number of fibroblasts (Fb), number of OCN-immunolabelled cells, content of collagen (CF), and intensity of inflammatory reaction in the capsules around the NeoSealer Flo (NeoFlo), Bio-C Sealer (BC), AH Plus (AHP) and Control group (CG) at 7, 15, 30 e 60 days of implantation.

Periods		NeoFlo	BC	AHP	CG
**7 days**	TC (µm)	360±46^a;1^	485±26^b;1^	349±65^a;1^	149±86^c;1^
	IC/mm^2^	1,570±24^a;1^	720±46^b;1^	797±73^b;1^	251±35^c;1^
	Fb/mm^2^	104±21^a;1^	128±21^a;1^	104±12^a;1^	168±10^a;1^
	CF (%)	13.4±5^a;1^	21.8±3^b;1^	3.4±2^c;1^	16.4±2^a;1^
	OCN/mm^2^	11.11(11.11)^a;1^	33.33(33.34)^b;1^	0.00 (0.0)^c;1^	0.00 (0.0)^c;1^
	Inflammatory reaction	intense	moderate	moderate	mild
**15 days**	TC (µm)	363±42^a;1^	386±31^a;1^	242±20^b;2^	189±11^c;1^
	IC/mm^2^	1,252±22^a;1^	579±27^b;2^	636±94^c;2^	231±15^d;1^
	Fb/mm^2^	151±21^a;1^	158±16^a;1^	202±21^b;2^	236±21^b;2^
	CF (%)	20.6±3^a;2^	22.8±2^a;1^	11.5±4^b;2^	28.9±3^a;2^
	OCN/mm^2^	22.22 (22.22)^a;2^	33.33(33.34)^b;1^	0.00 (0.0)^c;1^	0.00 (0.0)^c;1^
	Inflammatory reaction	moderate	moderate	moderate	mild
**30 days**	TC (µm)	281±47^a;2^	216±11^a;2^	229±24^a;2^	107±04^b,1^
	IC/mm^2^	933±67^a;2^	262±27^c;3^	453±39^b;3^	176±25^c,2^
	Fb/mm^2^	231±09^a;2^	221±9^a;2^	203±10^a;2^	310±23^b;3^
	CF (%)	25.8±1^a;3^	28.2±1^a;2^	20.9±3^a;3^	26.8±2^a;2^
	OCN/mm^2^	22.22 (22.22)^a;2^	44.44 (33.34)^b;2^	0.00 (0.0)^c;1^	0.00 (0.0)^c;1^
	Inflammatory reaction	moderate	mild	moderate	mild
**60 days**	TC (µm)	190±49^a;3^	146±6^a;3^	240 ± 43^b;2^	104 ± 20^a,1^
	IC/mm^2^	486 ± 40^a;3^	189±7^b;4^	352 ± 90^c;4^	68 ± 11^d,3^
	Fb/mm2	360±23^a;3^	329±27^a;3^	308 ± 12^a;3^	391 ± 23^b;3^
	CF (%)	29.9±2^a;3^	27.8±1^a;2^	23.8 ± 1^b;3^	29.4 ± 1^a;2^
	OCN/mm^2^	33.33(22.22)^a;3^	55.56 (33.34)^b;3^	0.00 (0.0)^c;1^	0.00 (0.0)^c;1^
	Inflammatory reaction	moderate	mild	moderate	mild

The comparison between groups in the same period is indicated by superscript letters in the lines; same letters = no significant difference.

The comparison between periods in the same group is indicated by superscript numbers in the columns; same numbers = no significant difference. Tukey test (p≤0.05). OCN: Values expressed as median and interquartile range. Analysis between groups in each period: Kruskal-Wallis followed by the Dunn test; analysis of each group over time: Friedman followed by the Nemenyi test (p<0.05).

### Inflammation reaction intensity

**Figure 3:**
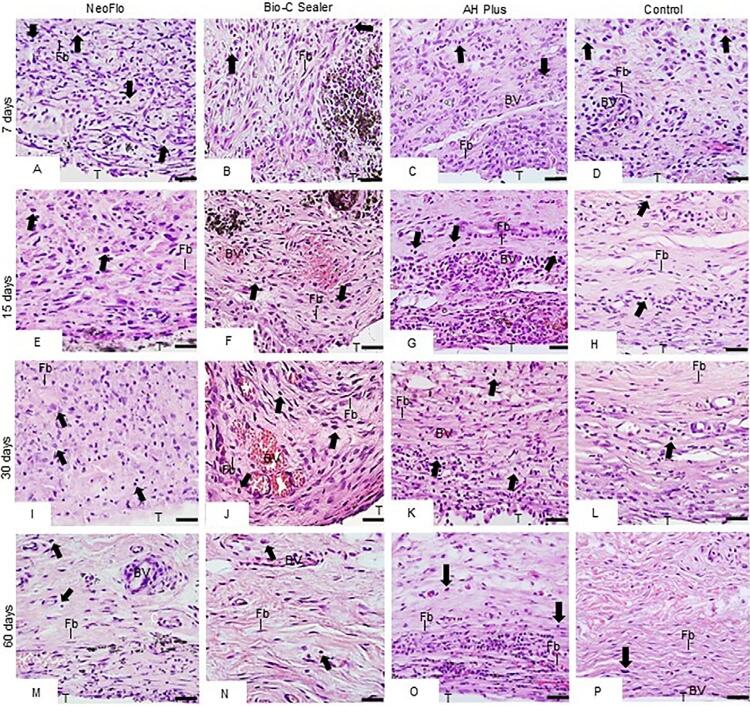
Photomicrographs of sections showing portions of capsules adjacent to the implanted tubes (T) after seven (A-D), 15 (E-H), 30 (I-L), and 60 days (M-P). Arrows, inflammatory cells; Fb, fibroblasts; CF, collagen fibers; BV, blood vessels. Bars: 18 μm.

The capsules around all specimens contained several IC and few collagen fibers at seven days (Figures 3A-3D). An intense inflammatory reaction was observed in the NeoFlo specimens, while the capsules showed a moderate inflammatory reaction in BC and AHP specimens (Table 1). A moderate inflammatory reaction was in the capsules adjacent to the materials after 15 days (Figure 3E-3G; Table 1). At 30 and 60 days, the capsules of NeoFlo (Figures 3I and 3M) and AHP (Figs. 3K and 3O) materials showed moderate inflammatory reaction (Table 1) while few IC were present (Figures 3J and 3N) in the BC, characterizing a mild inflammatory reaction (Table 1). A mild inflammatory reaction was seen in the capsules of CG specimens in all periods (Figures 3D, 3H, 3L, 3P and Table 1).

### Numerical density of ICs and Fb

The quantitative analyses (Table 1) revealed that the greatest values of IC were seen in the NeoFlo specimens in all time points. There was no significant difference in the number of IC between BC and AHP specimens at seven days (*p*>0.05), but the number of IC was significantly reduced in BC specimens in comparison with AHP at 15, 30, and 60 days (*p*<0.001). In all periods, there was no significant difference in the number of Fb among NeoFlo, BC, and AHP specimens (*p*>0.05), except at 15 days. The number of Fb was significantly greater in the AHP than in NeoFlo and BC specimens (*p*<0.05) in this period, but there was no significant difference between AHP and CG specimens (*p*>0.05). A significant reduction in the number of IC and a significant increase in the number of Fb was observed in all groups over time.

### Content of collagen in the capsules

The capsules contained few birefringent materials at seven days, which showed a significant increase over time (Table 1). The capsules around AHP showed the lowest values in all periods. Although the NeoFlo had lower birefringent collagen than in capsules of BC at seven days, no significant difference (*p*>0.05) was observed between two groups at 15, 30, and 60 days (Table 1).

### Immunohistochemical detection of OCN

**Figure 4:**
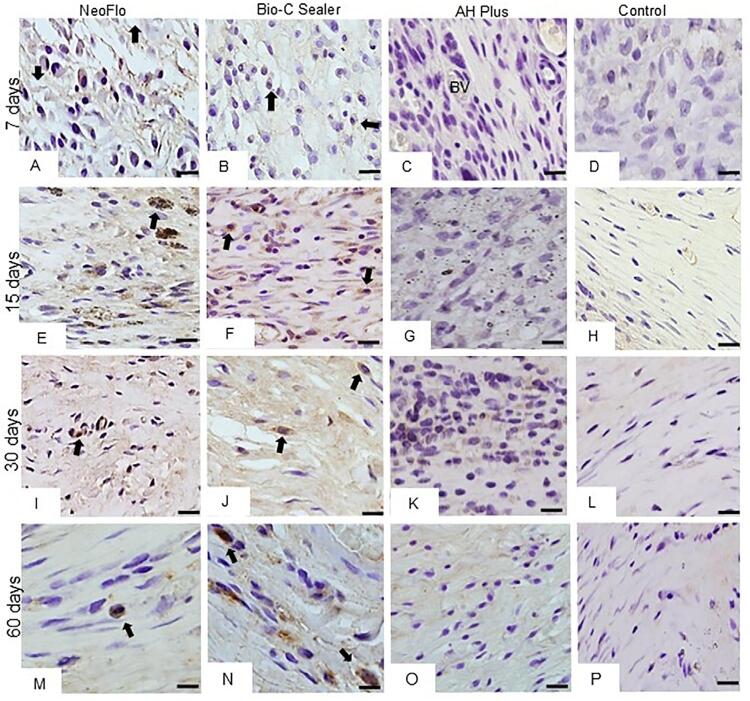
Photomicrographs of sections showing portions of capsules adjacent to the implanted tubes after seven (A-D), 15 (E-H), 30 (J-L), and 60 days (M-P). The sections were submitted to immunohistochemistry for OCN detection and counterstained with hematoxylin. OCN-immuno-abled cells (arrows) are seen in the capsules of NeoFlo and BC sealers. Bars: 18 μm.

OCN-immunolabelled cells were only observed in the capsules around NeoFlo and BC specimens (Figures 4A-4P). Table 1 shows that the number of OCN-immunolabelled cells was significantly greater in the BC specimens than in NeoFlo in all periods (*p*<0.05). From seven to 60 days, a significant increase in immunoexpression was observed in the capsules around NeoFlo and BC specimens.

### The von kossa reaction and analysis under polarized Light

**Figure 5:**
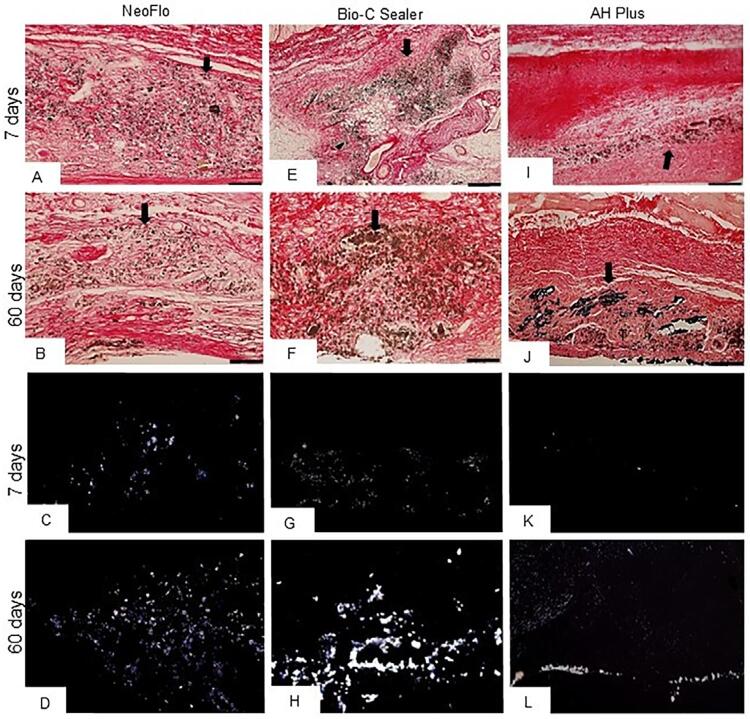
Photomicrographs of sections showing portions of capsules adjacent to the opening of the implanted tubes submitted to the von Kossa reaction (A-F) after seven and 60 days. Capsules of NeoFlo (A, D), BC (B, E), and AHP (C, E) show von Kossa-positive structures (black/brown color). Figures 5g-4l: unstained sections analyzed under polarized light. Birefringent structures are seen in the capsules. Bars: 36 μm.

The capsules around NeoFlo, BC, and AHP specimens had a von Kossa-positive structures in all periods (Figures 5A-5F). Birefringent structures were observed in the capsules around NeoFlo and BC specimens in all periods (Figures 5C, 5D, 5G and 5H). However, they were only seen in the innermost surface of the capsules in the AHP specimens (Figures 5I and 5L). The von Kossa-positive or birefringent structures were not observed in the CG specimens (data no shown).

## Discussion

The tissue reaction and bioactivity of NeoFlo were compared to BC and AHP after implantation in the subcutaneous connective tissue of rats. NeoFlo induced a greater number of inflammatory cells, maintaining moderate inflammatory reaction for up to 60 days. Nevertheless, NeoFlo could stimulate OCN immunoexpression demonstrating its bioactive potential. Therefore, the null hypothesis was rejected since the sealers showed different tissue reactions.

NeoFlo is a premixed bioceramic sealer composed of tricalcium silicate (<25%) and dicalcium silicate (<10%) as bioactive components, and calcium aluminate (<25%), calcium aluminum oxide (grossite) (<6%), tricalcium aluminate (<5%) and tantalite (<50%) as radiopacifier. NeoFlo has a thickening agent whose composition is not described by the manufacturer. The thickening agent may be related to the intense inflammatory reaction of NeoFlo in relation to the other sealers that promoted a moderate inflammatory process. This biological response may also be due to the high solubility of NeoFlo.^1^

3-(4,5-dimethylthiazol-2-yl)-2,5- diphenyltetrazolium bromide (MTT) analysis revealed that undiluted concentrations of NeoFlo were associated with a significant reduction in mitochondrial activity compared to the control group.^16^ Although calcium silicate-based sealers are generally considered cytocompatible, several authors have reported inconsistent results regarding cell viability, depending on the specific type of sealer used.^1,2,16^

A moderate inflammatory reaction was observed at 60 days, although a gradual reduction in the number of ICs around NeoFlo was observed over time. Nevertheless, the capsules around the BC cement showed a mild inflammatory reaction after 60 days. The initial inflammatory reaction induced by bioceramics cements occurs due to their high pH, which promotes the migration of ICs and the production of cytokines.^2^

Okamu, et al.^17^(2020) also observed excellent repair of periapical tissues from root canals filled with BC. López-García, et al.^18^(2019) demonstrated greater cytocompatibility and mineralization ability of the BC than AHP.

AHP is an endodontic sealer based on epoxy resin and is considered the gold standard due to its physical properties and high bond strength to dentin. Tolosa-Monfà, et al.^19^ (2023) demonstrated cytotoxicity caused by AHP and related to its composition. However, Silva, et al.^2^ (2020) demonstrated that this sealer is biocompatible in the subcutaneous tissue of rats after 60 days but does not have bioactive potential.

Endodontic sealers can release substances responsible for the inflammatory reaction in the connective tissue during their setting. The long setting time of NeoFlo (around 1,344 minutes)^1^ compared to BC (around 220 minutes)^7^ may also be responsible at least in part for difference in the inflammatory reaction intensity induced by these sealers.

In all groups, significant reduction in the number of ICs was observed with the gradual increase in the number of fibroblasts and content of birefringent collagen in the capsules. These findings support the concept that these endodontic sealers allow connective tissue repair.^15^ At 60 days, capsules around the NeoFlo and AHP specimens contain moderate inflammatory infiltrate and thicker capsules than those around BC specimens, indicating that NeoFlo and AHP exhibit greater irritant potential to connective tissue. The cytotoxicity of AHP has been related to the release of its amine and epoxy resin components.^20^ However, AHP demonstrates a reduction in the severity of inflammation over time.^21^

NeoFlo is composed of tricalcium silicate, dicalcium silicate, calcium aluminate, calcium aluminum oxide (grossite), tricalcium aluminate, and tantalite. Liu, et al.^22^ (2011) developed a sealer based on tricalcium aluminate and demonstrated that it had biocompatibility and bioactive potential. Calcium aluminate has three different phases, one of which is grossite. NeoMTA Plus contains tantalum oxide in its composition and exhibits biocompatibility and bioactive potential.^23^Tantalum oxide and tantalite are both based on tantalum; however, tantalite has a higher amount of tantalum, which can cause different tissue reactions. Sebastian, et al.^24^(2024) demonstrated that the biocompatibility of NeoFlo was acceptable against human gingival fibroblasts; however, it decreased over time.

Janini, et al.^25^ (2025) observed *in vivo* IL-6 expression predominantly in the perimaterial regions, with intense cellular immunostaining detected around NeoFlo a premixed material containing tantalum which also showed significant volumetric loss upon contact with tissues. However, NeoFlo was associated with the highest levels of zirconium accumulation in kidney samples. While tungsten accumulation was also notably elevated, tantalum in the premixed formulation of NeoFlo showed minimal systemic accumulation. Such results suggest that an ideal sealer composition has yet to be established, especially regarding local stability and systemic biocompatibility.

Neoputty is a repair material that has similar composition to NeoFlo. Lozzáno-Guillén, et al.^26^ (2020) demonstrated that periodontal ligament cells showed reduced cell viability when cultured with NeoMTA 2. Although Ca^2^ is an essential regulator of several intracellular processes, excessive intracellular Ca^2^ accumulation, and high alkalinity can promote mitochondrial dysfunction and, consequently, reduce cell viability.^27^

The evaluation of calcium-containing structure formation is obtained via histochemical techniques such as the von Kossa method, which is based on detecting calcium in tissue sections embedded in paraffin. This can be associated with immunohistochemical detection of non-collagenous proteins found in mineralized tissues, including osteocalcin (OCN), osteopontin, and osteonectin. Osteocalcin is a peptide secreted by osteoblasts and odontoblasts during the formation of the bone matrix and dentin matrix, respectively.^2,11,12^

Endodontic sealers based on calcium silicate are involved in osteoblast differentiation.^28^OCN is a small glycoprotein, which is preferentially expressed by osteoblasts, mainly in the late stages of their differentiation, and consequently its presence can indicate the ability to form mineralized tissue.^2,11,28-31^ OCN may indicate the activation of cells responsible for bone matrix synthesis and mineralization, which are essential aspects for evaluating the biocompatibility and potential of the material to promote bone regeneration.

OCN binds strongly to calcium and therefore seems to be involved in the regulation of matrix mineralization. OCN-immunolabelled cells were only observed in the capsules of NeoFlo and BC sealers in this study. BC showed the highest values of OCN-immunolabelled at 60 days. Thus, BC sealer can contribute to the mineralization of periapical tissues, since they demonstrate bioactive potential.^2^ Exacerbated inflammation can impair the activity of mesenchymal cells, such as fibroblasts and periodontal ligament stem cells, by reducing the expression of osteoblastic markers like OCN. Therefore, the more intense inflammatory response induced by NeoFlo may have disrupted the cellular signaling required to establish a microenvironment conducive to osteogenesis, which could explain the low OCN immunoexpression observed in this group.

According to Sanz, et al.^32^ (2024) Ta⁵⁺ was detected in NeoFlo, in accordance with the composition reported by the manufacturer. Differences in ion release may also influence the role of calcium silicates in the upregulation of mineralization-related gene expression by human periodontal ligament stem cells, which may explain the superior immunolabelling observed in capsules of BC.

In addition to the OCN immunoexpression, the capsules around these sealers also showed von Kossa-positive structures, which indicated the salt deposits (calcium and/or phosphate).^33^ There were birefringent structures only on the innermost surface of the capsules in the AHP specimens, in agreement with other studies that demonstrated lower release of calcium ions for AHP than promoted by other bioceramic sealers.^2,22,35^ Our von Kossa results combined with birefringent structures suggest a bioactive potential of NeoFlo like the BC sealer. The reaction process of calcium ions and carbon dioxide leads to the formation of calcite amorphous, a birefringent structure that is considered as a parameter suggestive of the bioactive potential of an endodontic material.^29,33,34^

Biocompatibility and bioactive potential are important properties for endodontic materials, since these materials can have direct contact with periapical tissues and interfere with the repair of periapical tissues, which is a process that aims at the success of endodontic treatment.^2,30-37^ Our findings confirm that NeoFlo showed inferior biocompatibility when compared to BC sealer.

## Conclusion

NeoFlo sealer induced a greater initial inflammatory response than BC and AHP. However, all materials showed a reduction in inflammatory cells and an increase in the number of fibroblasts and collagen organization over time. While NeoFlo showed low OCN immunoexpression in comparison to the BC, both sealers enabled amorphous calcite deposition, indicating a degree of bioactive potential. Such findings suggest that all evaluated materials demonstrated acceptable tissue responses over time, despite differences in inflammatory profile and bioactivity markers.

## References

[1] Zamparini F, Prati C, Taddei P, Spinelli A, Di Foggia M, Gandolfi MG (2022). Chemical-physical properties and bioactivity of new premixed calcium silicate-bioceramic root canal sealers. Int J Mol Sci.

[2] Silva EC, Tanomaru-Filho M, Silva GF, Delfino MM, Cerri PS, Guerreiro-Tanomaru JM (2020). Biocompatibility and bioactive potential of new calcium silicate-based endodontic sealers: Bio-C Sealer and Sealer Plus BC. J Endod.

[3] Camilleri J (2020). Classification of hydraulic cements used in dentistry. Front Dent Med.

[4] Benetti F, Gomes-Filho JE, Azevedo-Queiroz IO, Carminatti M, Conti LC, Reis-Prado AH (2021). Biological assessment of a new ready-to-use hydraulic sealer. Restor Dent Endod.

[5] Lepure C, Walsh RM, Attar S, Turner CL, Crawford J, Jalali P (2024). Clinical outcomes of nonsurgical root canal treatment using NeoSealer Flo and Endosequence BC Sealer: a retrospective analysis with short-term follow-up. Clin Oral Investig.

[6] Silva EC, Pradelli JA, Silva GF, Cerri PS, Tanomaru-Filho M, Guerreiro-Tanomaru JM (2025). Biocompatibility and bioactivity of bioceramic sealers containing 1% cetrimide. Braz Oral Res.

[7] Zordan-Bronzel CL, Torres FF, Tanomaru-Filho M, Chávez-Andrade GM, Bosso-Martelo R, Guerreiro-Tanomaru JM (2019). Evaluation of physicochemical properties of a new calcium silicate-based sealer, Bio-C Sealer. J Endod.

[8] Zhou H, Du T, Shen Y, Wang Z, Zeng Y, Haapasalo M (2015). In vitro cytotoxicity of calcium silicate-containing endodontic sealers. J Endod.

[9] Koutroulis A, Kuehne SA, Cooper PR, Camilleri J (2019). The role of calcium ion release on biocompatibility and antimicrobial properties of hydraulic cements. Sci Rep.

[10] Jung S, Libricht V, Sielker S, Hanisch MR, Schäfer E, Dammaschke T (2019). Evaluation of the biocompatibility of root canal sealers on human periodontal ligament cells ex vivo. Odontology.

[11] Delfino MM, Jampani JL, Lopes CS, Guerreiro-Tanomaru JM, Tanomaru-Filho M, Sasso-Cerri E (2023). The participation of fibroblast growth factor-1 and interleukin-10 in connective tissue repair following subcutaneous implantation of bioceramic materials in rats. Int Endod J.

[12] Delfino MM, Guerreiro-Tanomaru JM, Tanomaru-Filho M, Sasso-Cerri E, Cerri PS (2020). Immunoinflammatory response and bioactive potential of GuttaFlow bioseal and MTA Fillapex in the rat subcutaneous tissue. Sci Rep.

[13] de Pizzol JP, Sasso-Cerri E, Cerri PS (2018). Matrix metalloproteinase-1 and acid phosphatase in the degradation of the lamina propria of eruptive pathway of rat molars. Cells.

[14] Saraiva JA, Fonseca TS, Silva GF, Sasso-Cerri E, Tanomaru JMG, Tanomaru M (2018). Reduced interleukin-6 immunoexpression and birefringent collagen formation indicate that MTA Plus and MTA Fillapex are biocompatible. Biomedical Materials.

[15] Queiroz MB, Inada RN, Jampani JL, Guerreiro-Tanomaru JM, Sasso-Cerri E, Tanomaru-Filho M (2023). Biocompatibility, and bioactive potential of an experimental tricalcium silicate-based cement in comparison with Bio-C repair and MTA Repair HP materials. Int Endod J.

[16] López-García S, Sánchez-Bautista S, García-Bernal D, Lozano A, Forner L, Sanz JL (2024). Premixed calcium silicate-based ceramic sealers promote osteogenic/cementogenic differentiation of human periodontal ligament stem cells: a microscopy study. Microsc Res Tech.

[17] Okamura T, Chen L, Tsumano N, Ikeda C, Komasa S, Tominaga K (2020). Biocompatibility of a high-plasticity, calcium silicate-based, ready-to-use material. Materials.

[18] López-García S, Pecci-Lloret MR, Guerrero-Gironés J, Pecci-Lloret MP, Lozano A, Llena C (2019). Comparative cytocompatibility and mineralization potential of Bio-C Sealer and TotalFill BC. Sealer. Materials.

[19] Tolosa-Monfà A, Veroni A, Blasi-Cabús J, Ballester-Palacios ML, Berástegui-Jimeno E (2013). Cytotoxicity comparison of Bio C Sealer against multiple root canal sealers. J Clin Exp Dent.

[20] Giacomino CM, Wealleans JA, Kuhn N, Diogenes A (2019). Comparative biocompatibility and osteogenic potential of two bioceramic sealers. J Endod.

[21] Ashraf H, Shafagh P, Abbas FM, Heidari S, Shahoon H, Zandian A (2022). Biocompatibility of an experimental endodontic sealer (Resil) in comparison with AH26 and AH-Plus in rats: an animal study. J Dent Res Dent Clin Dent Prospects.

[22] Liu WN, Chang J, Zhu YQ, Zhang M (2011). Effect of tricalcium aluminate on the properties of tricalcium silicate-tricalcium aluminate mixtures: setting time, mechanical strength and biocompatibility. Int Endod J.

[23] Hoshino RA, Delfino MM, Silva GF, Guerreiro-Tanomaru JM, Tanomaru-Filho M, Sasso-Cerri E, Cerri PS (2021). Biocompatibility and bioactive potential of the NeoMTA Plus endodontic bioceramic-based sealer. Restor Dent Endod.

[24] Sebastian S, El-Sayed W, Adtani P, Zaarour RF, Nandakumar A, Elemam R (2024). Evaluation of the antibacterial and cytotoxic properties of TotalFill and NeoSEALER flo bioceramic sealers. J Conserv Dent Endod.

[25] Janini AC, Moraes BF, Pelepenko LE, Santos VA, Barros-Costa M, Malosá GF (2025). Physicochemical properties and biological interaction of calcium silicate-based sealers- in vivo model. Clinical Oral Investigations.

[26] Lozano-Guillén A, López-García S, Rodríguez-Lozano FJ, Sanz JL, Lozano A, Llena C (2022). Comparative cytocompatibility of the new calcium silicate-based cement NeoPutty versus NeoMTA Plus and MTA on human dental pulp cells: an in vitro study. Clin Oral Investig.

[27] Rodríguez-Lozano FJ, Lozano A, López-García S, García-Bernal D, Sanz JL, Guerrero-Gironés J (2022). Biomineralization potential and biological properties of a new tantalum oxide. Clin Oral Investig.

[28] Viola N, Guerreiro-Tanomaru J, Silva G, Sasso-Cerri E, Tanomaru-Filho M, Cerri PS (2012). Biocompatibility of an experimental MTA sealer implanted in the rat subcutaneous: quantitative and immunohistochemical evaluation. J Biomed Mater Res B Appl Biomater.

[29] Almeida LH, Moraes RR, Morgental RD, Pappen FG (2017). Are premixed calcium silicate-based endodontic sealers comparable to conventional materials? A systematic review of in vitro studies. J Endod.

[30] Silva EC, Tanomaru-Filho M, Silva GF, Lopes CS, Cerri PS, Guerreiro Tanomaru JM (2020). Evaluation of the biological properties of two experimental calcium silicate sealers: an in vivo study in rats. Int Endod J.

[31] Silva GF, Tanomaru-Filho M, Bernardi MI, Guerreiro-Tanomaru JM, Cerri PS (2015). Niobium pentoxide as radiopacifying agent of calcium silicate-based material: evaluation of physicochemical and biological properties. Clin Oral Investig.

[32] Sanz JL, López-García S, García-Bernal D, Rodríguez-Lozano FJ, Forner L, Lozano A (2024). Comparative bioactivity and immunomodulatory potential of the new Bioroot Flow and AH Plus Bioceramic sealer: an in vitro study on hPDLSCs. Clin Oral Investig.

[33] Bueno CR, Valentim D, Marques VA, Gomes-Filho JE, Cintra LT, Jacinto R (2016). Biocompatibility and biomineralization assessment of bioceramic-, epoxy-, and calcium hydroxide-based sealers. Braz Oral Res.

[34] Carvallo L, Henríquez B, Paredes R, Olate J, Onate S, Van Wijnen AJ (2008). 1a, 25-dihydroxy vitamin D3-enhanced expression of the osteocalcin gene involves increased promoter occupancy of basal transcription regulators and gradual recruitment of the 1a, 25-dihydroxy vitamin D3 receptor-SRC-1 coactivator complex. J Cell Physiol.

[35] Kapralos V, Böcker J, Vach K, Altenburger M, Proksch S, Karygianni L (2022). On the biocompatibility of endodontic sealers. Swiss Dent J.

[36] Donnermeyer D, Bürklein S, Dammaschke T, Schäfer E (2019). Endodontic sealers based on calcium silicates: a systematic review. Odontology.

[37] Silva GF, Guerreiro-Tanomaru JM, Fonseca TS, Bernardi MI, Sasso-Cerri E, Tanomaru-Filho M (2017). Zirconium oxide and niobium oxide used as radiopacifiers in a calcium silicate-based material stimulate fibroblast proliferation and collagen formation. Int Endod J.

